# Adaptive Individualized high-dose preoperAtive (AIDA) chemoradiation in high-risk rectal cancer: a phase II trial

**DOI:** 10.1007/s00259-022-05944-0

**Published:** 2022-09-21

**Authors:** Alessandra Guido, Dajana Cuicchi, Paolo Castellucci, Francesco Cellini, Francesca Di Fabio, Fabiola Lorena Rojas Llimpe, Lidia Strigari, Milly Buwenge, Savino Cilla, Francesco Deodato, Gabriella Macchia, Erika Galietta, Rita Golfieri, Andrea Ardizzoni, Rocco Maurizio Zagari, Stefano Fanti, Gilberto Poggioli, Lorenzo Fuccio, Alessio G. Morganti

**Affiliations:** 1 Radiation Oncology, IRCCS Azienda Ospedaliero-Universitaria di Bologna, Bologna, Italy; 2 Surgery of the Alimentary Tract, IRCCS Azienda Ospedaliero-Universitaria di Bologna, Bologna, Italy; 3 Nuclear Medicine, IRCCS Azienda Ospedaliero-Universitaria di Bologna, Bologna, Italy; 4 Dipartimento di Diagnostica per Immagini Radioterapia Oncologica ed Ematologia, Fondazione Policlinico Universitario “A. Gemelli” IRCCS, UOC di Radioterapia Oncologica, Roma, Italy; 5 Università Cattolica del Sacro Cuore, Dipartimento Universitario Diagnostica per immagini, Radioterapia Oncologica ed Ematologia, Largo Agostino Gemelli 8, 00168 Roma, Italy; 6 Medical Oncology, IRCCS Azienda Ospedaliero-Universitaria di Bologna, Bologna, Italy; 7 Department of Medical Physics, IRCCS Azienda Ospedaliero-Universitaria di Bologna, Bologna, Italy; 8 Department of Experimental, Diagnostic and Specialty Medicine-DIMES, Alma Mater Studiorum University of Bologna, Bologna, Italy; 9 Medical Physics, Gemelli Molise Hospital-Università Cattolica del Sacro Cuore, Campobasso, Italy; 10 Radiation Oncology, Gemelli Molise Hospital-Università Cattolica del Sacro Cuore, Campobasso, Italy; 11 Department of Radiology, IRCCS Azienda Ospedaliero-Universitaria di Bologna, Bologna, Italy; 12 IRCCS Azienda Ospedaliero-Universitaria di Bologna, Gastroenterology Unit, Department of Medical and Surgical Sciences, Gastroenterology Unit, University of Bologna, Bologna, Italy; 13 Department of Digestive Medicine and Surgery, Alma Mater Studiorum University of Bologna, Bologna, Italy

**Keywords:** Rectal neoplasms, Radiotherapy, Chemotherapy, Neoadjuvant, Preoperative, Intensity modulated, Simultaneous integrated boost, Adaptive, 18F-FDG-PET, Phase II

## Abstract

**Purpose:**

To evaluate the pathological complete response (pCR) rate of locally advanced rectal cancer (LARC) after adaptive high-dose neoadjuvant chemoradiation (CRT) based on ^18^ F-fluorodeoxyglucose positron emission tomography/computed tomography (^18^ F-FDG-PET/CT).

**Methods:**

The primary endpoint was the pCR rate. Secondary endpoints were the predictive value of ^18^ F-FDG-PET/CT on pathological response and acute and late toxicity. All patients performed ^18^ F-FDG-PET/CT at baseline (PET_0_) and after 2 weeks during CRT (PET_1_). The metabolic PET parameters were calculated both at the PET_0_ and PET_1_. The total CRT dose was 45 Gy to the pelvic lymph nodes and 50 Gy to the primary tumor, corresponding mesorectum, and to metastatic lymph nodes. Furthermore, a sequential boost was delivered to a biological target volume defined by PET_1_ with an additional dose of 5 Gy in 2 fractions. Capecitabine (825 mg/m^2^ twice daily orally) was prescribed for the entire treatment duration.

**Results:**

Eighteen patients (13 males, 5 females; median age 55 years [range, 41–77 years]) were enrolled in the trial. Patients underwent surgical resection at 8–9 weeks after the end of neoadjuvant CRT. No patient showed grade > 1 acute radiation-induced toxicity. Seven patients (38.8%) had *TRG* = 0 (complete regression), 5 (27.0%) showed *TRG* = 2, and 6 (33.0%) had *TRG* = 3. Based on the TRG results, patients were classified in two groups: *TRG* = 0 (pCR) and *TRG* = 1, 2, 3 (non pCR). Accepting *p* < 0.05 as the level of significance, at the Kruskal–Wallis test, the medians of baseline-MTV, *interim*-SUVmax, *interim*-SUVmean, *interim*-MTV, *interim*-TLG, and the MTV reduction were significantly different between the two groups. ^18^ F-FDG-PET/CT was able to predict the pCR in 77.8% of cases through compared evaluation of both baseline PET/CT and interim PET/CT.

**Conclusions:**

Our results showed that a dose escalation on a reduced target in the final phase of CRT is well tolerated and able to provide a high pCR rate.

## Introduction

Colorectal cancer is still one of the most prevalent cancers in industrialized countries and about one third of tumors are in the rectum [1]. Preoperative chemoradiation (CRT) is currently considered the standard treatment of locally advanced rectal cancer (LARC) due to the positive impact on loco-regional control and on probability of sphincter-sparing resection [2, 3].

Traditionally, preoperative CRT is based on the combination of 45 to 50.4 Gy delivered with conventional fractionation and concurrent fluoropirimidine-based chemotherapy. This approach achieved a complete pathological response (pCR) in up to 10–20% of patients [4, 5].

Intensity-modulated radiation therapy with simultaneous integrated boost (IMRT-SIB) technique is able to deliver the same doses to the prophylactic volumes plus a boost on the macroscopic disease in the same treatment session, with optimal sparing of the surrounding normal tissues. Therefore, IMRT-SIB technique is potentially associated with lower acute toxicity rates and thus to improved feasibility of dose-escalated CRT [6, 7].

It is worth noting that LARCs significantly shrink, in most cases, during CRT. Therefore, escalating the dose only to the residual gross target volume (GTV), at the end of CRT, could improve treatment tolerability and pCR rates and provide better chances of conservative surgery [8–10].

Adaptive radiotherapy is a technique based on the progressive conformation of the irradiated volumes during treatment. This approach was mainly tested in patients with head and neck cancers [11, 12], while reliable data on LARC are still lacking. Moreover, the possibility to achieve the same pCR rate as standard treatments, despite the reduction of treated volumes during CRT, was never proven. Furthermore, no data is available on the imaging technique of choice for early evaluation of tumor response.

^18^ F-FDG-PET/CT is not routinely used in the staging or tumor response assessment of LARCs [13]. However, some studies showed a high reliability of ^18^ F-FDG-PET/CT in predicting the pathological response after CRT [14–16].

Therefore, based on this background, aims of this study were to (i) evaluate pCR rates of LARC after adaptive high-dose neoadjuvant CRT with concomitant and sequential boost based on ^18^ F-FDG-PET/CT and (ii) confirm the value of ^18^ F-FDG-PET/CT in predicting the pCR rate.

## Material and methods

### Study design and aims

This was a prospective phase II study approved by the local ethics committee and registered in an international public registry (NCT03479814). All patients enrolled in the study signed an informed consent.

**Fig. 1:**
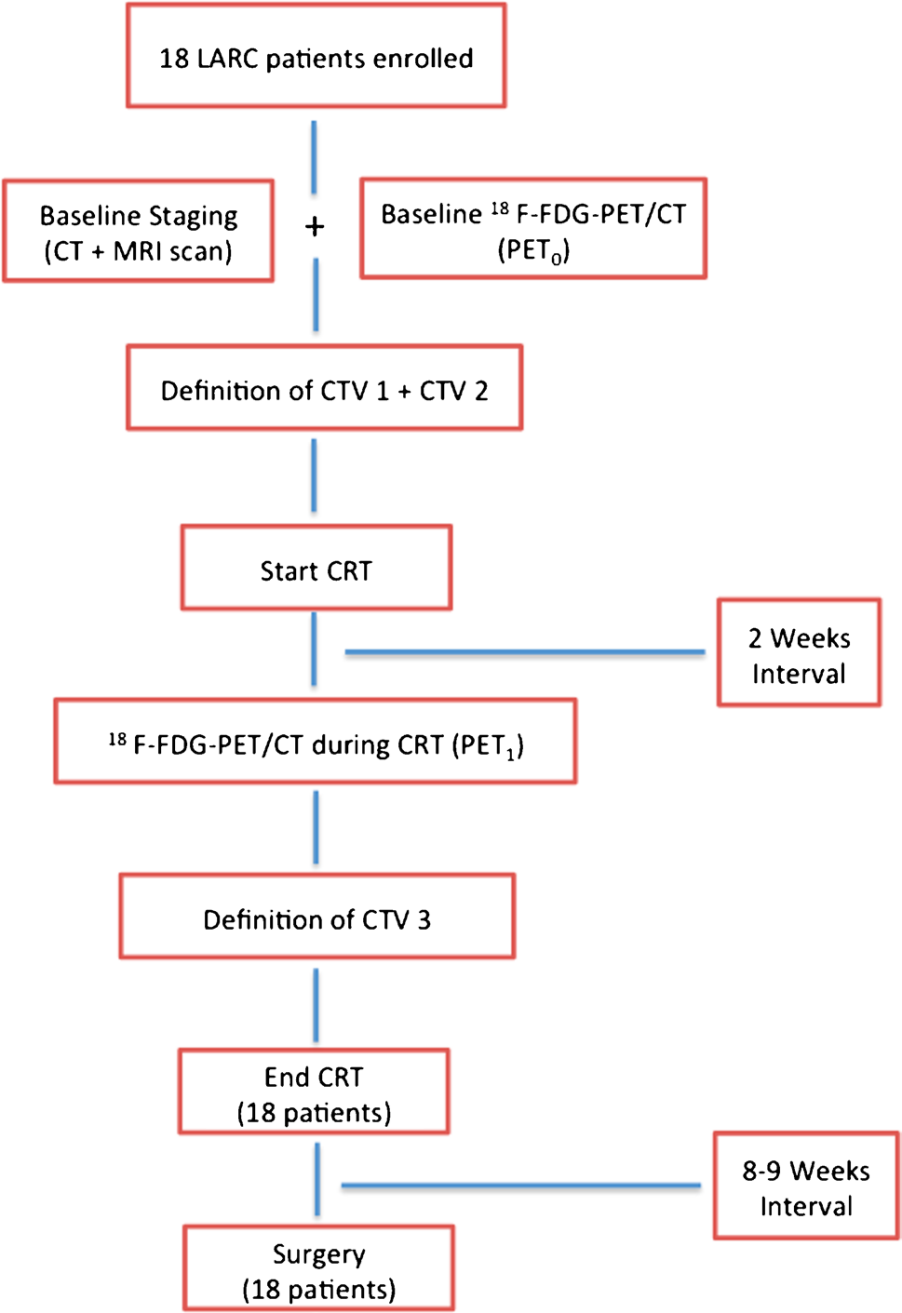
Flow chart of the study

The primary aim was to assess the pCR rate after ^18^ F-FDG-PET/CT-based neoadjuvant CRT. Secondary aims were as follows: (a) treatment-related acute and late toxicity, (b) GTV-boost and PTV-boost reduction through ^18^ F-FDG-PET/CT re-evaluation 2 weeks after starting CRT, (c) predictive value of ^18^ F-FDG-PET/CT on pathological response, (d) progression-free survival (PFS), (e) overall survival (OS), and (f) treatment impact on quality of life (QoL). A flow chart of the trial is summarized in Fig. 1.

### Eligibility criteria

Inclusion criteria were biopsy proven LARC with cT3-4N0-2M0 (any tumor site) or T2N1-2 M0 (only lower rectum) stage; age ≥ 18 years; Eastern Cooperative Oncology Group performance status ≤ 2; adequate hematological count and liver/renal function. Exclusion criteria were as follows: patients unfit for chemotherapy or surgery, metastatic disease not amenable for local radical treatments, pregnant or breast-feeding, severe cardiovascular disease, prior pelvic radiotherapy, patients with other primary neoplasms (except non-melanoma skin cancer or in situ cervical carcinoma), and patients not able to provide informed consent.

### Outcome measures

At baseline, a clinical evaluation based on rectal examination and complete clinical history was performed. Subsequent assessments included colonoscopy with biopsy, routine blood tests with carcino-embryonic antigen (CEA), liver and renal function, trans-rectal ultrasound (TRUS), contrast-enhanced thorax-abdomen-pelvis computed tomography (CT) scan, and pelvic magnetic resonance imaging (MRI).^18^ F-FDG-PET/CT was performed before the start (PET_0_) and 2 weeks after starting CRT (PET_1_) to plan the sequential boost.

Clinical response was assessed with clinical examination, contrast-enhanced thorax-abdomen-pelvis CT scan, and pelvic MRI. Surgery was planned about 8 weeks after CRT completion.

Pathological tumor response was scored according to the College of American Pathologists [17] as follows: tumor regression grade (TRG)-0: non-viable cancer cells (complete response); TRG-1: single cells or rare small groups of cancer cells (near complete response); TRG-2: residual cancer with evident tumor regression but more than single cells or rare small groups of cancer cells (partial response); TRG-3: extensive residual cancer with no evident tumor regression (poor or no response). Based on the TRG results, patients were classified in two groups: *TRG* = 0 (complete pathological response) and *TRG* = 1, 2, 3 (non-complete pathological response).

### Radiotherapy planning

All patients were immobilised with full bladder in supine position using the Combifix™ frame and underwent a baseline ^18^ F-FDG-PET/CT scan. Whole-body^18^ F-FDG-PET/CT was performed using a standard procedure. Briefly, 3.0 MBq/kg of ^18^F-FDG was intravenously injected. All patients were required to fast for 6 h and the uptake time was 60 min. Images were acquired on a 3-D tomograph (Discovery STE; GE) for 2 min per bed position. A low-dose CT scan (120 kV, 80 mA) was performed both for attenuation correction and to provide an anatomical map. ^18^ F-FDG-PET/CT images were reconstructed using an iterative 3-D ordered subsets expectation maximization method with two iterations and 20 subsets, followed by smoothing (with a 6-mm 3-D Gaussian kernel) with CT-based attenuation, scatter, and random coincidence event correction [18].

### PET-CT analysis and target volumes definition

All ^18^ F-FDG-PET/CT scans were reviewed by two experienced nuclear medicine physicians who defined the PET positive regions. Sites of primary tumor and metastatic lymph nodes were defined as the GTV-PET_0_. A radiation oncologist with over 10-year experience in LARC treatment defined two clinical target volumes (CTV1 and CTV2). The CTV1 included the GTV-PET_0_ and the corresponding mesorectum (same cranio-caudal level) plus 2 cm cranio-caudally. The CTV2 included the CTV1 plus the entire mesorectum, the pre-sacral space, the internal iliac nodes, and the obturator nodes. Inguinal nodes were included in case of positive inguinal nodes or in patients with infiltration of the anal canal and/or external anal sphincter, and/or lower third of the vagina. The planning target volumes (PTV1 and PTV2) were generated by adding an isotropic expansion of 0.8 cm from CTV1 and CTV2, respectively.

**Fig. 2:**
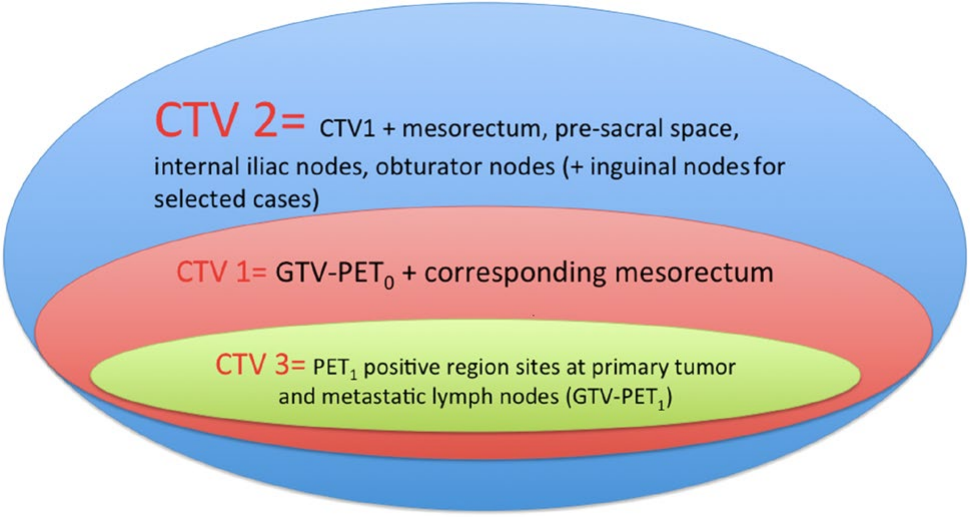
CTV definition

Two weeks after CRT start, an interim ^18^F-FDG-PET/CT (PET_1_) was performed to plan the second phase of the treatment (sequential boost). Two nuclear medicine physicians examined the interim ^18^F-FDG-PET/CT (PET_1_), compared it with the PET_0_, and finally defined the PET_1_ positive region sites at primary tumor and metastatic lymph nodes as the GTV-PET_1_. Then, the radiation oncologist defined the CTV of the sequential boost (CTV3) as the GTV-PET_1_ plus an isotropic expansion of 0.5 cm. The PTV3 was generated by isotropically adding 0.8 cm to the CTV3. Figure 2 depicts the CTV delineation.

### Image analysis and interpretation criteria

The following metabolic ^18^F-FDG-PET/CT parameters of LARCs were measured both at baseline and at the interim PET: SUVmax (maximum standardized uptake value), SUVmean (mean standardized uptake value), MTV (metabolic tumor volume), and TLG (total lesion glycolysis). MTV measurement was calculated on ^18^F-FDG-PET/CT images using a semi-quantitative (40% threshold) analysis. When necessary a visual evaluation was added to the semi quantitative analysis to avoid missing the tumor at the boundaries, SUVmax and SUVmean, normalized to body weight, were calculated within the MTV defined as above. TLG values were calculated as the product of MTV and SUVmean [19]. When the bladder (filled with radioactive urine) was very close to the primary lesion, a visual correction of the adjacent region of interest margin was necessary. Moreover, we calculated the SUVmax, SUVmean, MTV, and TLG percentage reduction between the baseline and interim scan using the following formula:

$$\frac{(baseline\;scan\;value-interim\;scan\;value)}{baseline\;scan\;value}*100$$

### Organs at risk (OARs)

The following OARs were considered: bowel (defined as the “bowel bag”), bladder, and femoral heads. The acceptability of dose distribution to the OaRs was evaluated based on the dose/volume constraints suggested by the quantitative analysis of normal tissue effects in the clinic (QUANTEC) guidelines [20].

### Intensity-modulated RT (IMRT)

IMRT was delivered using an Elekta Sinergy Linac (Elekta, Crowley, United Kingdom), equipped with standard multi leaf collimators, with 6–15 MV photon energy. During the first phase of CRT (IMRT-SIB on PTV1 and PTV2), a daily online check of the set-up was performed using an electronic portal imaging device, as previously described [21]. During the sequential boost (PTV3), before each daily session, patients underwent KV-Cone-Beam-CT to check and eventually correct organ motion and set-up inaccuracies. The RT dose delivered to the PTV2 was 45 Gy (1.8 Gy/fraction) with 50 Gy (2 Gy/fraction) SIB dose to the PTV1 in five consecutive days per week. The dose delivered to the PTV3 was 5.0 Gy (2.5 Gy/die) on two consecutive days (PTV3 total dose: 55 Gy). Planning and delivery processes underwent a systematic independent-check procedures, as previously described [22]. In patients with grade ≥ 3 acute toxicity, CRT was stopped until toxicity was decreased to at least grade 2 toxicity.

### Chemotherapy

Concurrent chemotherapy was based on capecitabine (825 mg/m^2^ twice daily orally) and was prescribed for the entire CRT duration. The choice of adjuvant chemotherapy was left at the medical oncologist’s discretion based on initial stage and pathological examination.

### Surgery

Surgery was scheduled about eight weeks after the end of the CRT. The total mesorectal excision with pelvic autonomic nerve preservation was performed when technically feasible. However, the choice among surgical approaches (abdomino-perineal resection or low anterior resection) was based on physical examination and the results of pelvic MRI-based restaging.

### Follow-up

The first follow-up visit included physical examination and full blood count and was performed 4 weeks after surgery. Chest CT scan and abdominal-pelvic CT scan or MRI were performed every 6 months in the first 5 years and yearly thereafter. Local control was calculated from the date of diagnosis to the time of local–regional failure or last follow-up. Disease-free survival was defined as the time from diagnosis to local or distant recurrence or last follow-up. Overall survival was defined as the time from diagnosis until death from any cause or last follow-up. Acute and late toxicity data were scored according to the Common Terminology Criteria for Adverse Events (CTCAE v4.03). Quality of Life was evaluated using the EORTC QLQ-C30 questionnaire at the beginning and at the end of radiotherapy.

### Sample size and statistical analysis

According to the Simon’s optimal two-stage design [23], this study required the enrolment of nine to 17 patients to prove or exclude a significant improvement of pCR rates. We planned the closure of the trial in case of no pCR in the first nine patients, while, in case of at least one pCR, the study was continued by including eight additional patients. However, considering a possible 5% drop-out rate, we increased the sample size of the second step to nine patients. Due to the small sample size, we used both the non-parametric Kruskal–Wallis test to compare the medians of ^18^F-FDG-PET/CT parameters and the Student’s *t*-test to compare the means of the same variables between the two groups (*TRG* = 0 versus* TRG* = 1, 2, 3) [24]. The univariate logistic regression was used to investigate whether metabolic PET parameters may predict the TRG [25]. Statistical analyses were performed using SPSS 20 (IBM Corp., Armonk, NY).

## Results

Between September 2017 and October 2018, eighteen patients were enrolled in the trial (13 males, 5 female; median age: 55 years, range: 41–77 years). Tumor site was the inferior, middle, and superior rectum in 10 (55%), six (33%), and two (11%) patients, respectively. According to the American Joint Committee on Cancer (AJCC 2010), the clinical stages were T2N1M0 (2 patients), T3N1M0 (11 patients), T3N2M0 (2 patients), and T4N1M0 (3 patients). The mean GTV-PET_0_ and GTV-PET_1_ values were 21.97 cc (SD: ± 24.32) and 9.76 cc (SD: ± 15.26), respectively (*p* = 0.002). The mean PTV2 and PTV3 values were 175.6 cc (SD: ± 81.4) and 41.1 cc (SD: ± 36.5), respectively (*p* < 0.001).

Nineteen episodes of G1 acute toxicity were recorded in 17 patients (gastrointestinal: 7, genitourinary: 7, hematological: 2, skin: 3) while no patient showed acute G 2 toxicity. One patient with dihydro-pyrimidine dehydrogenase deficiency had acute G3 toxicity (hematological and gastrointestinal) while no patient showed *G* > 3 acute toxicity. Based on the EORTC QLQ-C30 questionnaire, no patient had relevant (> 20) changes in terms of quality of life. No patient showed late toxicity during the follow-up.

Surgical resection was performed eight-nine weeks after the end of CRT. Low anterior resection was performed in 14 patients (77%), abdomino-perineal resection in three patients (16%), and local excision in one patient (5.5%) who refused radical surgery after achieving a clinical complete response. According to the College of American Pathologists, seven patients (38.8%) had *TRG* = 0; five patients (27%) *TRG* = 2 and six patients (33%) had TRG 3.

**Fig. 3:**
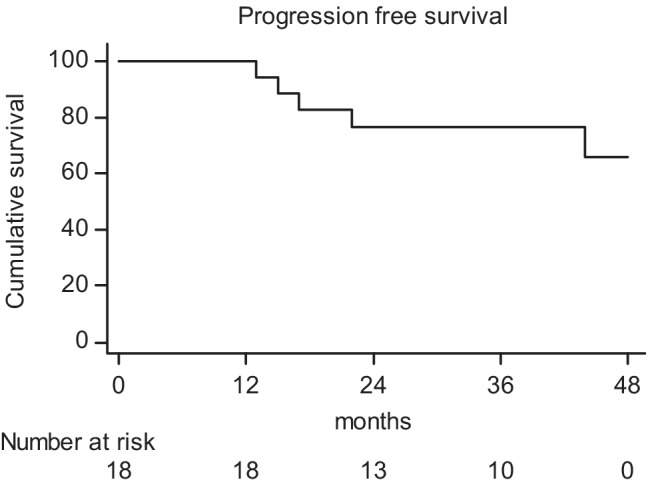
Actuarial progression-free survival

**Fig. 4:**
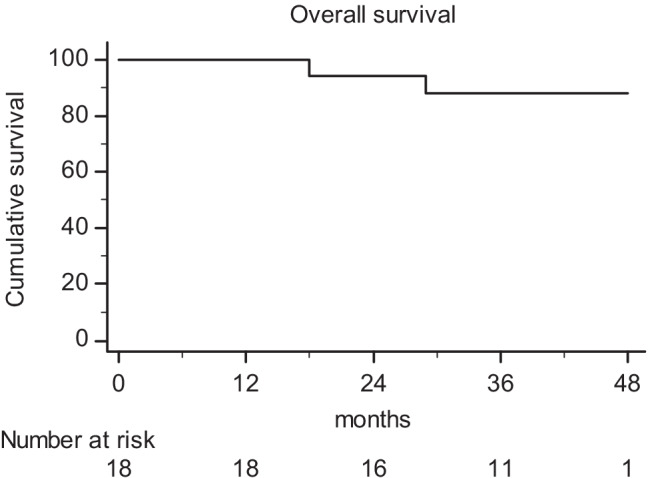
Actuarial overall survival

The median follow-up was 41.5 months (range: 13.0–50.0 months). Three patients showed hematogenous metastases, while two had distant metastases and synchronous local recurrence. The latter were both males, with low rectal cancer, and clinical stage T4N1. One-, 2-, 3-, and 4-year PFS rates were 100%, 76.7%, 76.7%, and 65.8%, respectively (Fig. 3). Finally, 1-, 2-, 3-, and 4-year OS rates were 100%, 94.1%, 88.2%, and 88.2%, respectively (Fig. 4).

At Kruskal–Wallis test, the median values of baseline-MTV, *interim*-SUVmax, *interim*-SUVmean, *interim*-MTV, *interim*-TLG, and the MTV reduction were significantly different between the group with *TRG* = 0 and the group with *TRG* = 1, 2, 3 (*p* = 0.016, *p* = 0.013, *p* = 0.030, *p* = 0.006, *p* = 0.006, and *p* = 0.033, respectively). Moreover, at the Student’s *t*-test, the means of the same variable reported above were significantly different between the two groups (*p* = 0.007, *p* = 0.025, *p* = 0.027, *p* = 0.007, *p* = 0.008, and *p* = 0.021, respectively). The results of the Kruskal–Wallis test and the Student’s *t*-test are presented in Tables 1 and 2, respectively.

**Table 1:** Nonparametric Kruskal–Wallis test — median values in *TRG* = 0 versus *TRG* = 1, 2, 3

Parameters	*TRG* = 0 (median)	*TRG* = 1, 2, 3 (median)	*p*-value
SUVmax — baseline	11.8	12.9	0.298
SUVmean — baseline	7.3	8.0	0.618
MTV — baseline	6.1	24.1	**0.016**
TLG — baseline	44.5	213.2	0.063
SUVmax — ad interim	5.3	9.7	**0.013**
SUVmean — ad interim	4.3	6.1	**0.030**
MTV — ad interim	2.4	15.4	**0.006**
TLG — ad interim	6.2	95.5	**0.006**
SUVmax reduction (%)	56.3	31.7	0.113
SUVmean reduction (%)	41.0	26.2	0.258
MTV reduction (%)	64.7	27.8	**0.033**
TLG reduction (%)	79.1	46.1	0.052

Texts in bold font style highlights values with statistical significance

*Legend*: *TRG*, tumor regression grade; *SUVmax*, maximum standardized uptake value; *SUVmean*, mean standardized uptake value; *MTV*, metabolic tumor volume; *TLG*, total lesion glycolysis

**Table 2:** Student’s *t*-test — mean values in *TRG* = 0 versus* TRG* = 1, 2, 3

Parameters	*TRG* = 0 (mean ± *SD*)	TRG = 1, 2,3 (mean ± *SD*)	*p*-value
SUVmax — baseline	12.1 (± 4.8)	17.5 (± 11.1)	0.238
SUVmean — baseline	7.3 (± 3.1)	8.9 (± 4.7)	0.432
MTV — baseline	7.6 (± 4.5)	23.9 (± 16.0)	**0.007**
TLG — baseline	65.9 (± 256.7)	65.8 (± 256.7)	0.075
SUVmax — ad interim	5.6 (± 4.3)	11.0 (± 4.6)	**0.025**
SUVmean — ad interim	3.8 (± 2.3)	6.3 (± 2.0)	**0.027**
MTV — ad interim	2.186 (± 2.0)	17.164 (± 14.6)	**0.007**
TLG — ad interim	11.6 (± 14.7)	116.9 (± 105.2)	**0.008**
SUVmax reduction (%)	49.9 (± 38.1)	30.1 (± 22.2)	0.178
SUVmean reduction (%)	41.3 (± 39.0)	22.4 (± 25.6)	0.229
MTV reduction (%)	67.0 (± 29.5)	33.1 (± 26.1)	**0.021**
TLG reduction (%)	71.3 (± 36.2)	47.4 (± 25.2)	0.116

Texts in bold font style highlights values with statistical significance

*Legend*: *TRG*, tumor regression grade; *SUVmax*, maximum standardized uptake value; *SUVmean*, mean standardized uptake value; *MTV*, metabolic tumor volume; *TLG*, total lesion glycolysis

All variables were tested with a univariate logistic regression analysis to evaluate their role as TRG predictors. The only independent predictor of TRG was the MTV reduction between baseline- and *interim-*PET (odds ratio = 1.048, 95% CI = 1.001–1.097, Nagelkerke R2 = 0.378; *p* = 0.045). The logistic model built with this regressor correctly predicts the TRG after CRT in 77.8% of patients. Independent variables are reported in terms of odds ratios (OR) and their 95% confidence intervals (CI) in Table 3.

**Table 3:** Univariate logistic regression: association between PET parameters and TRG

PET parameters	*p* =	Exp (B)	95% CI for Exp (B)	
			Lower	Upper
SUVmax — baseline	0.273	1.112	0.920	1.345
SUVmean — baseline	0.422	1.128	0.841	1.513
MTV — baseline	0.058	1.186	0.994	1.415
TLG — baseline	0.097	1.011	0.998	1.025
SUVmax — interim	0.066	1.435	0.977	2.109
SUVmean — interim	0.065	2.044	0.957	4.366
MTV — interim	0.075	1.487	0.960	2.304
TLG — interim	0.088	1.052	0.992	1.116
SUVmax reduction (%)	0.184	1.027	0.988	1.067
SUVmean reduction (%)	0.232	1.022	0.986	1.060
MTV reduction (%)	**0.045**	1.048	1.001	1.097
TLG reduction (%)	0.127	1.031	0.991	1.072

Texts in bold font style highlights values with statistical significance

## Discussion

The achievement of pCR after CRT is an important predictor of improved long-term outcome in LARC patients [26]. Standard CRT delivered with conventional doses (45–50 Gy) induces up to 20% pCR rates. RT dose intensification based on the delivery of SIB [27] as well as adaptive strategies [28] was investigated in order to improve these figures and clinical outcome in LARC.

To the best of our knowledge, this is the first report on ^18^ F-FDG-PET/CT -based adaptive dose-escalated CRT in LARC. Main aim of the current study was to evaluate the pathological response of LARC after adaptive high-dose neoadjuvant CRT with both simultaneous and sequential boost planned based on ^18^ F-FDG-PET/CT. We recorded 38.8% pCR rate with a low incidence of severe toxicity. In fact, only one case of grade 3 diarrhea was registered, yielding a severe toxicity rate of 5.5%.

The findings of our study are noteworthy for at least three reasons. First of all, we showed that ^18^ F-FDG-PET/CT was able to predict pCR in more than 75% of cases. Secondly, the implementation of adaptive CRT by ^18^ F-FDG-PET/CT allowed the delivery of dose-escalated treatment without worsening acute and late toxicity. Arguably, the dramatic reduction in PTV (by 76.6%), made possible by the interim ^18^ F-FDG-PET/CT, contributed to this result. Finally, this approach increased the pCR rate up to about 40% of cases.

^18^ F-FDG-PET/CT, as well as MRI, can be theoretically used for adaptive dose-escalated CRT in LARC [29–, 30–32]. Moreover, some studies reported a significant correlation between early ^18^ F-FDG-PET/CT and pathological tumor response [33–35]. Furthermore, the use of ^18^ F-FDG-PET/CT has been studied to optimize the initial target volume in preoperative CRT of LARC [34]. In the literature, the values of SUVmax have been found to correlate with response to CRT in rectal cancer [36]; interestingly, in our experience, the strongest prognosticator is the metabolic tumor volume reduction from the baseline to the interim ^18^ F-FDG-PET/CT.

In fact, in the prospective study by Alongi et al. [34], SIB-based dose intensification was tested in patients with LARC using ^18^ F-FDG-PET/CT. The latter was performed before CRT and was merged with the planning-CT scan to define a high-dose volumes including the hyper-metabolic areas of the primary tumor and metastatic nodes. Sixty and 54 Gy were delivered in 30 fractions to the hyper-metabolic areas and to the prophylactic volume, respectively. Tumor downstaging was reported in 62.5% of cases but the pCR rate was only 17.5%. Furthermore, ^18^ F-FDG-PET/CT was not able to predict pCR and no correlation was found between pre-treatment SUV-max values and pCR. However, unlike in our study, ^18^ F-FDG-PET/CT was carried out only before and not during CRT. This could explain the different results about pCR rate and ^18^ F-FDG-PET/CT predictive value.

Furthermore, as mentioned above, an interim ^18^ F-FDG-PET/CT, showing a rapid reduction of LARCs during CRT [37], has the further advantage of reducing the OaR-irradiated volume in the final phase of the treatment, with improved feasibility of intensified CRT regimens. Obviously, the greatest concern regarding dose-escalated CRT in LARC patients is the increased risk of toxicity, particularly in terms of gastrointestinal adverse effects. Some prospective studies based on IMRT (+ / − SIB), but without an adaptive strategy, have shown 23–35% pCR and 5–27% grade ≥ 3 toxicity rates [26, 27]. Our combined approach allowed to further improved pCR but without worsening of toxicity. This has important clinical implication based on the emerging data on the possibility to avoid major surgery in LARC patients with complete clinical response after preoperative CRT [30, 38].

The main limitations of our study are both small sample size and lack of a control group. Nevertheless, this trial should be considered as an exploratory study since it is the first prospective test of early ^18^ F-FDG-PET/CT to allow CRT dose escalation in LARC. Based on the results of our study, we can speculate that higher doses may be tested through this combined ad interim ^18^ F-FDG-PET/CT-based approach.

In conclusion, the results of our trial showed that adaptive individualized high-dose neoadjuvant CRT delivered with simultaneous and sequential boosts planned with ^18^ F-FDG-PET/CT is feasible and effective. In fact, our study showed that dose-escalation in the final phase of CRT is well tolerated and able to provide high pCR rate with a favorable toxicity profile.

## Abbreviations

CRT: Chemoradiation
LARC: Locally advanced rectal cancer
pCR: Pathological complete response
IMRT-SIB: Intensity modulated radiation therapy with simultaneous integrated boost
GTV: Gross target volume
^18^ F-FDG-PET/CT: ^18^ F-fluorodeoxyglucose positron emission tomography/computed tomography
PFS: Progression-free survival
OS: Overall survival
QoL: Quality of life
CEA: Carcino-embryonic antigen
TRUS: Trans-rectal ultrasound
CT: Contrast-enhanced thorax-abdomen-pelvis computed tomography
MRI: Pelvic magnetic resonance imaging
PET_0_: ^18^ F-FDG-PET/CT at baseline
PET_1_: ^18^ F-FDG-PET/CT after 2 weeks during CRT
TRG: Tumor regression grade
CTV: Clinical target volume
PTV: Planning target volume
SUVmax: Maximum standardized uptake value
SUVmean: Mean standardized uptake value
MTV: Metabolic tumor volume
TLG: Total lesion glycolysis
OARs: Organs at risk
IMRT: Intensity-modulated RT
QUANTEC: Quantitative analysis of normal tissue effects in the clinic
CTCAE: Common Terminology Criteria for Adverse Events
AJCC: American Joint Committee on Cancer
